# Broccoli Fluorets: Split Aptamers as a User-Friendly Fluorescent Toolkit for Dynamic RNA Nanotechnology

**DOI:** 10.3390/molecules23123178

**Published:** 2018-12-02

**Authors:** Morgan Chandler, Tatiana Lyalina, Justin Halman, Lauren Rackley, Lauren Lee, Dylan Dang, Weina Ke, Sameer Sajja, Steven Woods, Shrija Acharya, Elijah Baumgarten, Jonathan Christopher, Emman Elshalia, Gabriel Hrebien, Kinzey Kublank, Saja Saleh, Bailey Stallings, Michael Tafere, Caryn Striplin, Kirill A. Afonin

**Affiliations:** 1 Nanoscale Science Program, Department of Chemistry, University of North Carolina at Charlotte, Charlotte, NC 28223, USA; mchand11@uncc.edu (M.C.); jhalman@uncc.edu (J.H.); laur3n_rackl3y@yahoo.com (L.R.); llee48@uncc.edu (L.L.); ddang6@uncc.edu (D.D.); wke@uncc.edu (W.K.); ssajja17@vt.edu (S.S.); swoods17@uncc.edu (S.W.); sachary3@uncc.edu (S.A.); elib4x4@gmail.com (E.B.); jchris67@uncc.edu (J.C.); eelshali@uncc.edu (E.E.); ghrebien@uncc.edu (G.H.); kkublan2@uncc.edu (K.K.); ssaleh6@uncc.edu (S.S.); bstalli4@uncc.edu (B.S.); mtafere@uncc.edu (M.T.); cdstripl@uncc.edu (C.S.); 2 Laboratory of Solution Chemistry of Advanced Materials and Technologies, ITMO University, Lomonosova St. 9, 191002 St. Petersburg, Russia; lyalina.at@gmail.com

**Keywords:** RNA nanotechnology, aptamer, Broccoli, dynamic nanoparticles, conditional activation

## Abstract

RNA aptamers selected to bind fluorophores and activate their fluorescence offer a simple and modular way to visualize native RNAs in cells. Split aptamers which are inactive until the halves are brought within close proximity can become useful for visualizing the dynamic actions of RNA assemblies and their interactions in real time with low background noise and eliminated necessity for covalently attached dyes. Here, we design and test several sets of F30 Broccoli aptamer splits, that we call fluorets, to compare their relative fluorescence and physicochemical stabilities. We show that the splits can be simply assembled either through one-pot thermal annealing or co-transcriptionally, thus allowing for direct tracking of transcription reactions via the fluorescent response. We suggest a set of rules that enable for the construction of responsive biomaterials that readily change their fluorescent behavior when various stimuli such as the presence of divalent ions, exposure to various nucleases, or changes in temperature are applied. We also show that the strand displacement approach can be used to program the controllable fluorescent responses in isothermal conditions. Overall, this work lays a foundation for the future development of dynamic systems for molecular computing which can be used to monitor real-time processes in cells and construct biocompatible logic gates.

## 1. Introduction

DNA and RNA, which are best known for their roles in processing genetic information, are natural biopolymers that are also increasingly utilized in the design and construction of nucleic acid nanoparticles (NANPs) [1,2,3,4]. Predictable pairing between canonical Watson–Crick bases (A–U (or T) and G–C) in RNA and DNA as well as the possibility for additional non-canonical base pairs, generally characteristic of RNA [5], allow for the assembly of programmable and well-defined structures that can coordinate an array of functions [6]. Such functions can be demonstrated by the diverse roles of natural nucleic acids (mostly RNA) as exemplified by riboswitches, ribozymes, mRNAs, aptamers, and siRNAs that are capable of fine-tuning and orchestrating biological environments [6]. RNA nanotechnology aims to design functional NANPs based on RNA’s ability to self-assemble, interact with other molecules, and exhibit fine-tunable physicochemical properties [2,7,8,9]. Rationally designed nucleic acids which are programmed to interact with other sequences, molecules, or in response to various stimuli have led to the development of molecular logic gates and biosensors [10,11,12,13,14,15]. The most recent achievements take advantage of these properties in constructing NANPs which are dynamic in structure and can independently [16,17] or interdependently [18,19] act in human cells, conditionally activating pre-programmed functionalities and triggering responses. Similar design principles can be applied to engineer diagnostic devices and smart therapeutics [20]. The rapid development of NANP-based dynamic platforms demands synchronized advancements in various robust visualization and tracking techniques that are user-friendly and biocompatible.

The responsive behaviors of NANPs can be directly visualized in living cells using complementary strands labeled with pairs of dyes that can undergo Förster resonance energy transfer (FRET). Tracing changes in FRET signals that result upon re-hybridization of labeled strands entering the compositions of cognate NANPs confirms the dynamicity of their behavior in real time [18,21,22]. The integrity of NANPs in cells can be verified through the co-localization of multiple different fluorophores simultaneously entering the composition of NANPs [23,24]. RNA probes also allow for a modular approach to visualization; fluorescent in situ hybridization, or FISH, and molecular beacons have been introduced to cells to give a fluorescent response upon binding to a target sequence [25,26,27]. However, for all aforementioned techniques, the fluorescent dyes must be covalently linked to either the 5′- or 3′-end of nucleic acids, which makes the techniques limited to only exogenously introduced NANPs.

For visualizing intracellular RNAs, the MS2-green fluorescent protein (GFP) system has been utilized to tag and image endogenous RNAs in cells [28]. However, this approach requires tagging RNAs with multiple copies of GFP systems in order to separate the desired signal from the background noise. This may affect the mobility of labeled RNAs and drastically alter their function. In turn, the split GFP system, in which two halves of the GFP are brought within binding proximity for restored fluorescence, has allowed for a conditionally activated fluorescence signal [18] and this approach can potentially be used to validate the formation of NANPs in cells [29,30]. The combination of several fluorescent proteins forming FRET pairs is another possible way for NANP visualizations [31]. However, all these systems require the presence of bulky tags and may be limited to the intracellular compartmentalization of NANPs.

The development of RNA aptamers which activate fluorophores upon binding has been a major improvement over these imaging techniques, offering a high signal-to-noise ratio, modularity for simple sequence incorporation and tagging, and real-time protein-free imaging in cells [25]. Using systematic evolution of ligands by exponential enrichment, or SELEX, to select a unique RNA sequence which binds and activates a normally non-fluorescent dye, the malachite green (MG) aptamer was first developed [32]. Similar to split protein systems, splitting MG aptamers allowed for fluorescence detection of nucleic acids [33,34]. In nucleic acid nanotechnology, any fluorescent aptamers can act as functional units which can be easily embedded into the NANPs’ structures by simple extension of individual strands. By doing this, NANPs’ assembly verification [35], tracking NANPs’ co-transcriptional assembly [7,35], and monitoring the dynamic behavior of interdependent RNA-DNA hybrids [22,36] have been successfully achieved. However, the high cytotoxicity of MG (a triphenylmethane dye) and its non-specific binding to cellular components warranted the further search for new biocompatible RNA aptamers which could be used at higher concentrations in cells [25]. Using SELEX, a Spinach aptamer was selected to bind a GFP fluorophore analog, the dye (*Z*)-4-(3,5-difluoro-4-hydroxybenzylidene)-1,2-dimethyl-1*H*-imidazol-5(4*H*)-one (DFHBI), and to exhibit green fluorescence when bound [37,38]. The Spinach aptamer was further optimized into Spinach2 for greater thermostability and brightness, yet still required a tRNA scaffold to promote folding and stability which made it susceptible to endonucleases and limited cellular activity [39,40]. A new aptamer with a three-way junction scaffold called F30-Broccoli was subsequently developed with a higher T_m_ and higher binding affinity for ligand DFHBI-1T, an optimized dye [25,40,41,42].

Fluorescent RNA aptamers such as Spinach, Broccoli, and Mango have been used to monitor a variety of metabolites and proteins in mammalian and bacterial cells [38,43,44,45,46]. To assess the actions of dynamic NANPs, these new aptamers were also split such that fluorescence is restored only upon the subsequent halves of the aptamer being brought into close proximity to re-associate and bind to a dye. Split Spinach [47] and split Broccoli [18] have been developed as tools for assessing the conditional activation of fluorescence. Based on the studied G-quadruplex structure of the Spinach aptamer which is involved in binding DFHBI, the sequence of Spinach was shortened into Baby Spinach while exhibiting comparable fluorescence [48]. As the Broccoli aptamer is also expected to depend on a G-quadruplex for fluorescence activation, we set out to optimize the split F30-Broccoli aptamer experimentally in order to produce several conditionally activated splits, that here we call fluorets, without the complexities of solving for multiple cocrystal structures of Broccoli bound to DFHBI-1T [40,48]. We suggest several experimental schemes allowing for both conditional activation and deactivation of fluorescent responses using the split aptamers technology.

## 2. Results and Discussion

**Figure 1:**
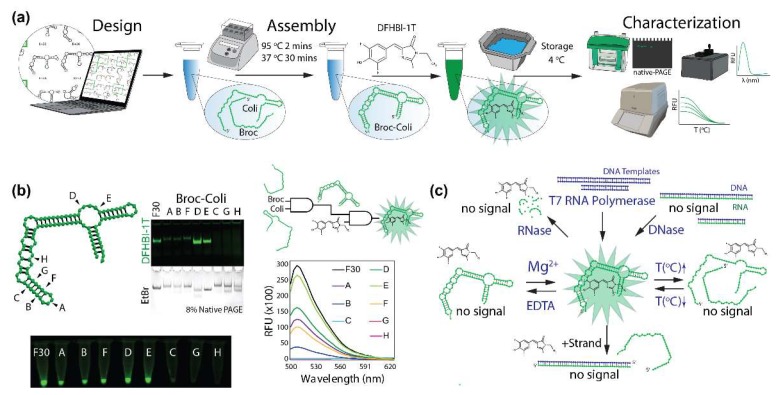
Computer-aided design, assembly, and characterization of fluorets. (**a**) Schematic representation of experimental pipeline with used experimental techniques indicated. (**b**) Positions of cuts (denoted A–H) chosen to be tested in this work and assessment of functionalities with native-PAGE and fluorimetry. Conditional activation of fluorescence is schematically demonstrated by AND gates. (**c**) Schematic representation of conditional activation and deactivation of fluorets tested in this work.

Rationally designed (as described in Methods) split F30-Broccoli (or original F30-Broccoli) aptamers were assembled in the presence of DFHBI-1T to assess their responses to an array of stimuli (Figure 1a) which would allow for their optimized utilization as tools in RNA nanotechnology. Potential folding and assembly of fluorets were assessed using NUPACK [49] (SI Figure S1) and from this library, eight different variations of the split F30-Broccoli aptamer were experimentally tested for assembly and fluorescence activation by non-denaturing polyacrylamide gel electrophoresis (native-PAGE) and fluorescence measurements. The results revealed that out of eight of the chosen fluorets, only five exhibited detectable fluorescence (Figure 1b). Because the fluorets have an additional starting sequence necessary for synthesis by in vitro transcription, their assemblies appear slightly higher on the gel when compared to the complete F30 aptamer. Fluorets D and E which appear to fluoresce the brightest in the presence of DFHBI-1T do not assemble; the Broc half of each fluoret fluoresces on its own, as the G-quadruplex DFHBI-1T-binding structure is not affected by the location of the split (SI Figure S2). This suggests using the individual Broc strands of D and E as truncated versions of F30 Broccoli. Once the active fluorets were identified, we then tested various stimuli to conditionally activate or deactivate them (Figure 1c). We proposed several important stimuli such as the presence of enzymes (RNase, DNase, or T7 RNA polymerase), responses to changes in temperature, presence of divalent ions, and programmability via the addition of complementary strands. Developing molecular devices able to respond to these stimuli may potentially assist in studies of metabolic processes and the dynamic behavior of NANPs.

**Figure 2:**
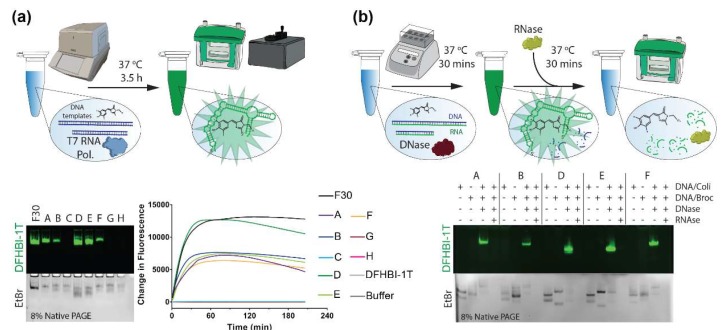
Enzyme-assisted activation and deactivation of fluorescent responses. (**a**) Co-transcriptional assembly of fluorets in the presence of DFHBI-1T. (**b**) DNase-assisted production of active fluorets from RNA/DNA duplexes, and their further deactivation with RNases.

We first experimentally confirmed that fluorets can be assembled co-transcriptionally while activating DFHBI-1T fluorescence. We followed the protocols of previously established generalized in vitro methodology for the one-pot T7 RNA polymerase-driven co-transcriptional assembly of different RNA NANPs [35], including those functionalized with up to ten siRNAs for co-RNAi [50]. In vitro run-off transcription performed with the mixture of DNA duplexes carrying T7 RNA polymerase promoters resulted in relatively high NANP assembly yields. In this work, using DNA templates of complementary fluorets in the presence of T7 RNA polymerase and transcription mix, complete fluoret RNAs were transcribed and assembled over 3.5 h, visualized by native-PAGE, and tracked in real-time using fluorescence (Figure 2a). The results suggest this system for further investigation with NANP production in cells. The ability to transcribe and assemble NANPs co-transcriptionally in mammalian cells is a promising avenue for their applications in vivo and assemblies with incorporated fluorets would allow for their simple visualization [35]. The use of mammalian cell lines for large-scale NANP production is expected to reduce endotoxin contamination and pave the way towards NANP-based personalized therapeutics. T7 RNA polymerase, which can be expressed in mammalian cells [51,52], would provide tight control over transcription regulation (cytoplasmic polymerase with unique 20 bps promoter), permits the use of shorter DNA templates, and offers faster transcription rates compared to RNA polymerase II.

It is beneficial to trace the activity and involvement of various enzymes in real time. As a proof of concept, we tested if the presence of different nucleases can conditionally drive either assembly or disassembly of fluorescent fluorets. Hybrid DNA/RNA duplexes for each fluoret monomer with an excess of DNA were incubated with DNase, resulting in the assembly of complete RNA fluorets which were then further deactivated by RNase (Figure 2b). The increased chemical stability of RNA/DNA hybrids [22] offers a way to keep fluorets dormant (thus, extending their shelf-life) until activation is desired.

**Figure 3:**
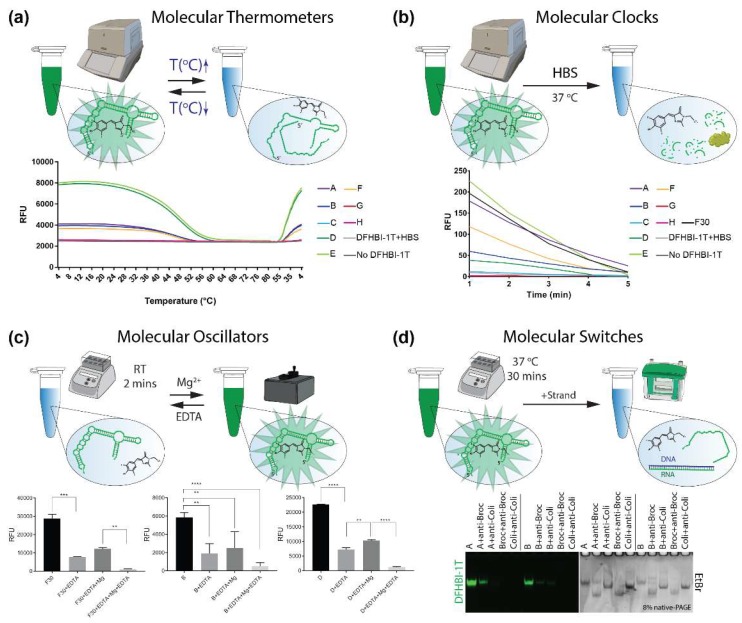
Molecular devices built with fluorets. (**a**) “Molecular Thermometer” that tracks the temperature changes via fluorescence activation and deactivation. (**b**) “Molecular Clock” that can trace the presence of sample in human blood serum via the fluorescence deactivation. (**c**) “Molecular Oscillators” working in response to the presence of magnesium ions. Statistical analysis was performed by one-way ANOVA (** *p* < 0.01, *** *p* < 0.001, **** *p* = 0.0001). (**d**) “Molecular Switches” responding to the introduction of oligonucleotides.

The temperature-responsive behavior of fluorets may enable them to act as “molecular thermometers” in that their fluorescence can be turned “off” and “on” again upon reaching a specific melting temperature (Figure 3a and SI Figure S3). This responsiveness allows for a personalized selection of the splits to be incorporated into NANPs based on their T_m_s (SI Table S1) and the temperature of the environment in which they will be functioning. The fluorets also demonstrate some minor variations in stability in human blood serum (Figure 3b), allowing them to act as “molecular clocks” for tracking in circulation during in vivo studies. The thermal and chemical stability of fluorets can be extended by potentially elongating their flanking helices or changes in Mg^2+^ concentrations. Oscillations between the activation and deactivation of the fluorescence can not only be controlled with temperature, but also upon the addition of a chelating agent such as ethylenediaminetetraacetic acid (EDTA) that removes the Mg^2+^ ions necessary for forming the tertiary structure of the aptamers required for binding and activating DFHBI-1T fluorescence. Importantly, the functional structure can be restored upon the subsequent addition of Mg^2+^ and the oscillation can continue based on repeated additions (Figure 3c).

Logic gating and the development of programmable NANPs often utilize strand displacement for triggering dynamic interactions [53]. Likewise, the fluorets can act as “molecular switches” which are activated or de-activated by the addition of other oligonucleotides [54]. Complementary DNAs to the split Broc or Coli strands which contain programmable toeholds can be used to disassemble fluorets and can in turn be displaced by full complement strands for the re-assembly of the fluoret with restored fluorescence (Figure 3d). Single stranded fluorets D and E can be deactivated by adding the complementary strands. This isothermal strand displacement is thermodynamically driven and becomes possible due to the presence of unpaired nucleotides in the secondary structure of F30-Broccoli as well as T7 RNA polymerase starting sequences added to the 5′-ends of all fluorets. The deactivation of F30-Broccoli, however, requires at least five-fold excess of complementary strand (SI Figure S5). Fluorets as output strands in molecular circuits can be used to provide a fluorescent response only when both are produced. While current applications for logic gating remain in vitro, future work will characterize the brightness of fluorets in various cell lines separately and when incorporated into dynamic NANP assemblies [55] for their transition to in vivo work.

## 3. Materials and Methods

### 3.1. Design of Broccoli Fluorets

Starting with the original split F30-Broccoli aptamer [18] with a cut after U45 separating Broc (45 nts) from Coli (72 nts), the fluorets were designed by simply moving the location of the split downstream 3 bases or upstream 3 bases. The +/− number indicates how many bases away from the original split site the new cut was made (e.g., B + 6 is 51 nts long, Coli − 6 is 66 nts long). The final group of eight fluorets was chosen via the analysis of NUPACK [49] predicted secondary structures (SI Figure S1), looking for either the greatest similarity to the original F30-Broccoli aptamer or for structures which addressed the importance of various functional regions in the aptamer sequence. With the original split [18] labeled as A, splits B, C, F, G and H were selected to be within close proximity in order to elucidate the relationship between the structure of the hairpin containing the original split and DFHBI-1T binding. Splits D and E were chosen to test the truncated versions of the F30-Broccoli scaffold.

### 3.2. RNA Preparation

DNA strands were purchased from Integrated DNA Technologies (Coralville, IA, USA) (Sequences available in Supporting Information) and PCR-amplified using MyTaq™ Mix from Bioline (London, UK). PCR products containing T7 RNA polymerase promoters were purified using the DNA Clean and Concentrator™ kit from Zymo Research (Irvine, CA, USA). RNAs were produced by in vitro run-off transcription with T7 RNA polymerase (80 mM HEPES-KOH (pH 7.5), 2.5 mM spermidine, 50 mM DTT, 25 mM MgCl_2_, 5 mM each rNTP). After 3.5 h at 37 °C, the reaction was incubated with RQ1 RNase-free DNase (New England BioLabs, Ipswich, MA, USA) prior to purification using denaturing 8 M urea polyacrylamide gel electrophoresis (PAGE, 15%). RNA bands were visualized under UV (short wavelength), cut, and eluted in crush and soak buffer (300 mM NaCl, 89 mM tris-borate (pH 8.2), 2 mM EDTA) overnight. Precipitation of the RNA was done in 2.5 volumes of 100% ethanol for 3 h at −20 °C. Samples were rinsed with 90% ethanol, vacuum dried, and dissolved in double-deionized water (17.8 MΩ·cm).

### 3.3. Broccoli Aptamer and Fluoret Assembly

F30-Broccoli RNA or Broc and Coli RNA strands composed of unmodified nucleotides were mixed in an equimolar ratio in double-deionized water. The samples were heated to 95 °C for 2 min, snap-cooled to 4 °C for 2 min, and assembly buffer (89 mM tris-borate (pH 8.2), 2 mM MgCl_2_, 50 mM KCl) was added, followed by 30 min of incubation at 37 °C.

### 3.4. Co-Transcriptional Assembly

PCR-amplified DNA templates for complementary splits were added 1:1 as 20% final volume with T7 RNA polymerase (80 mM HEPES-KOH (pH 7.5), 2.5 mM spermidine, 50 mM DTT, 25 mM MgCl_2_, 5 mM each rNTP) and 10 µM DFHBI-1T in buffer. Samples in triplicate were placed at 37 °C on a Bio-Rad CFX96 Touch Real-Time PCR Detection System (Bio-Rad, Hercules, CA, USA) for 3.5 h. Fluorescence was measured every minute, using 10 µM DFHBI-1T in buffer and transcription reactions at the above concentrations but without pDNA as negative controls.

### 3.5. Electrophoretic Mobility Shift Assays

For analysis, 8% non-denaturing native-PAGE (37.5:1) was used in the presence of 89 mM tris-borate (pH 8.2) and 2 mM MgCl_2_. Native-PAGE gels were run for 20 min (Mini-PROTEAN^®^ Tetra system by Bio-Rad, Hercules, CA, USA) at 4 °C and 300 V. Prior to staining, gels were washed 3 × 5 min in water and stained for 15 min in 10 µM DFHBI-1T in buffer (50 mM HEPES (pH 7.4), 100 mM KCl, 1 mM MgCl_2_) as previously reported [40]. Gels were imaged using a ChemiDoc MP system (Bio-Rad) for Alexa488. Afterwards, gels were washed again with water and stained for 10 min with ethidium bromide for total visualization.

### 3.6. EDTA Degradation and Mg^2+^ Formation

Fluorets were assembled as normal at a concentration of 4 µM with 10 µM DFHBI-1T in buffer and measured using a NanoDrop 3300 (Thermo Scientific, Waltham, MA, USA). An amount of 1 µL of 10 mM EDTA was added to the sample, which was incubated at room temperature for 2 min before measuring again with a NanoDrop 3300. This was repeated using 1 µL of 10 mM Mg^2+^ and incubating for 2 min at room temperature and again using 10 mM EDTA.

### 3.7. Nuclease-Driven Assembly/Degradation

Split Broc RNA strands were assembled with Coli DNA strands (and vice-versa) by mixing in an equimolar ratio in double-deionized water. The samples were heated to 95 °C for 2 min, snap-cooled to 4 °C for 2 min, and assembly buffer (89 mM tris-borate (pH 8.2), 2 mM MgCl_2_, 50 mM KCl) was added, followed by 20 min of incubation at room temperature. Each hybrid split was then added with its opposite hybrid split (e.g., dBroc + rColi was added with rBroc + dColi) in an equimolar ratio at a final concentration of 1 µM with 2/15 volume of RQ1 RNase-free DNase (New England BioLabs). This was incubated at 37 °C for 30 min, then 2/5 of the volume were added to RNase ONE™ (Promega, Madison, WI, USA) at a volume equal to the RQ1 RNase-free DNase added previously. This was incubated at 37 °C for 30 min. Samples were visualized on an 8% non-denaturing native-PAGE (37.5:1) in the presence of 89 mM tris-borate (pH 8.2) and 2 mM MgCl_2_ run for 20 min at 4 °C and 300 V.

### 3.8. Strand Displacement

Fluorets were assembled as previously described and then added in an equimolar ratio with either DNA for Broc or DNA for Coli prior to incubation at 37 °C for 30 min. RNA fluoret monomers were also assembled with their complementary DNAs as controls by mixing in an equimolar ratio in double-deionized water, heating to 95 °C for 2 min, snap-cooling to 4 °C for 2 min, and adding assembly buffer (89 mM tris-borate (pH 8.2), 2 mM MgCl2, 50 mM KCl) followed by 20 min of incubation at room temperature.

### 3.9. Thermal Deactivation/Activation

Aptamers were assembled as previously described. For in vitro staining with DFHBI-1T, assemblies were mixed in a 1:1 volumetric ratio with 20 µM DFHBI-1T in buffer (100 mM HEPES (pH 7.4), 200 mM KCl, 2 mM MgCl_2_) and incubated for 30 min at 37 °C. Assemblies at a final concentration of 1.25 µM underwent a 4 °C to 80 °C thermal gradient with a step size of 0.5 °C per 5 s using a Bio-Rad CFX96 Touch Real-Time PCR Detection System. For the reactivation of fluorets, assemblies were programmed to undergo cooling from 80 °C to 4 °C with a step size of 2 °C per 45 s. Fluorescence of thermal deactivation was measured in triplicate for all fluorets using 10 µM DFHBI-1T in buffer and F30-Broccoli without DFHBI-1T (1.25 µM) as negative controls. Analysis of T_m_ ± SEM was done in GraphPad Prism Software (Version 7, GraphPad Software, San Diego, CA, USA) with a Boltzmann sigmoidal curve fit.

### 3.10. Blood Stability

Aptamers were assembled as previously described, mixed in a 1:1 volumetric ratio with 20 µM DFHBI-1T in buffer, and incubated for 30 min at 37 °C. An amount of 9 µL of assembled aptamer at a final concentration of 1.25 µM was added with 1 µL of 20% HBS and placed at 37 °C on a Bio-Rad CFX96 Touch Real-Time PCR Detection System. Fluorescence was measured every min, using 2% HBS with 10 µM DFHBI-1T in buffer and 1.25 µM of F30-Broccoli with 2% HBS without DFHBI-1T as negative controls. Analysis of T_1/2_ was done in GraphPad Prism Software with a linear fit.

### 3.11. Statistics

Statistical analysis was done by one-way analysis of variance (ANOVA) using GraphPad Prism Software. All column means were compared by Tukey’s multiple comparison test. A *p*-value of less than 0.05 was considered to be statistically significant.

## 4. Conclusions

In conclusion, the experimental design reported in this work is anticipated to lead the development of several robust strategies allowing for real-time fluorescence-assisted tracking of various processes at the nanoscale level. The reported system is made of biocompatible materials that can be used for a broad range of biological and nanotechnological applications both in vitro and potentially in vivo. The developed experimental schemes are expected to help address some fundamental questions such as co-transcriptional folding of RNAs, formation of multi-stranded RNA NANPs, and their responses to various stimuli. Such complex behaviors will definitely promote fields such as synthetic biology or applications such as the stimulation of differentiating cells during basic research and tissue engineering. Although only eight different variations in the split aptamer were tested, optimized fluorescence of the split was demonstrated and shown to be responsive to various stimuli.

## Supplementary Materials

The following are available online, Figure S1: Secondary structures of designed Broccoli Fluorets; Figure S2: Fluorescence of individual monomer strands; Figure S3: “Molecular Thermometers” that track the temperature changes via fluorescence deactivation; Figure S4: “Molecular Switches” responding to the introduction of oligonucleotides; Figure S5: molecular switching of F30-Broccoli; Figure S6: Effect of [Mg^2+^] on fluorescence responses. Table S1: Physicochemical characterization of experimentally tested F30-Broccoli fluorets.
