# Supplementary material for: Broccoli Fluorets: Split Aptamers as a User-Friendly Fluorescent Toolkit for Dynamic RNA Nanotechnology
Source: Molecules. 2018 Dec 2;23(12):3178. doi: 10.3390/molecules23123178 (PMC6321606; doi:10.3390/molecules23123178)
Supplement: Supplementary file 1 [file molecules-23-03178-s001.pdf]

Supporting Information:

Broccoli Fluorets: Split Aptamers as a User-Friendly Fluorescent Toolkit for Dynamic RNA Nanotechnology

Morgan Chandler [1], Tatiana Lyalina [2], Justin Halman [1], Lauren Rackley [1], Lauren Lee [1], Dylan Dang [1], Weina Ke [1], Sameer Sajja [1], Steven Woods [1], Shrija Acharya [1], Elijah Baumgarten [1], Jonathan Christopher [1], Emman Elshalia [1], Gabriel Hrebien [1], Kinzey Kublank [1], Saja Saleh [1], Bailey Stallings [1], Michael Tafere [1], Caryn Striplin [1] and Kirill A. Afonin [1,*]

[1]Nanoscale Science Program, Department of Chemistry, University of North Carolina at Charlotte, Charlotte NC 28223, USA

[2] Laboratory of Solution Chemistry of Advanced Materials and Technologies, ITMO University, Lomonosova St. 9, 191002 St. Petersburg, Russian Federation

*To whom correspondence should be addressed: Kirill A. Afonin
Phone: +1 704 687 0685, Fax: +1 704 687 0960, Email: kafonin@uncc.edu

Sequences Designed in this Project

(Sequences in green were tested experimentally and all nucleotides are unmodified)

Cut ID	Sequences (5'-3')	Delta G Free energy (kcal/mol)
F30 Broccoli	GGGAAAGUUGCCAUGUGUAUGUGGGAGACGGUCGGGUCCAGAUAUUCGUAUCUGUCGAGUAGAGUGUGGGCUCCCACAUACUCUGAUGAUCCUUCGGGAUCAUUCAUGGCAA	-42.50
+0	Broc:<br>GGGAAAUUGCCAUGUGUAUGUGGGAGACGGUCGGGUCCAGAUAUU<br>Coli:<br>GGGAAACGUAUCUGUCGAGUAGAGUGUGGGCUCCCACAUACUCUGAUGAUCCUUCGGGAUCAUUCAUGGCAA	-44.78
+3	Broc+3:<br>GGGAAAUUGCCAUGUGUAUGUGGGAGACGGUCGGGUCCAGAUAUUCGU<br>Coli-3:<br>GGGAAAAUCUGUCGAGUAGAGUGUGGGCUCCCACAUACUCUGAUGAUCCUUCGGGAUCAUUCAUGGCAA	-46.08
+6	Broc+6:<br>GGGAAAUUGCCAUGUGUAUGUGGGAGACGGUCGGGUCCAGAUAUUCGUAUC<br>Coli-6:<br>GGGAAAUGUCGAGUAGAGUGUGGGCUCCCACAUACUCUGAUGAUCCUUCGGGAUCAUUCAUGGCAA	-42.98
+9	Broc+9:<br>GGGAAAUUGCCAUGUGUAUGUGGGAGACGGUCGGGUCCAGAUAUUCGUAUCUGU<br>Coli-9:<br>GGGAAACGAGUAGAGUGUGGGCUCCCACAUACUCUGAUGAUCCUUCGGGAUCAUUCAUGGCAA	-47.28
+12	Broc+12:<br>GGGAAAUUGCCAUGUGUAUGUGGGAGACGGUCGGGUCCAGAUAUUCGUAUCUGUCGA<br>Coli-12:<br>GGGAAAGUAGAGUGUGGGCUCCCACAUACUCUGAUGAUCCUUCGGGAUCAUUCAUGGCAA	-46.28
+15	Broc+15:<br>GGGAAAUUGCCAUGUGUAUGUGGGAGACGGUCGGGUCCAGAUAUUCGUAUCUGUCGAGUA<br>Coli-15:<br>GGGAAAGAGUGUGGGCUCCCACAUACUCUGAUGAUCCUUCGGGAUCAUUCAUGGCAA	-47.28
+18	Broc+18:<br>GGGAAAUUGCCAUGUGUAUGUGGGAGACGGUCGGGUCCAGAUAUUCGUAUCUGUCGAGUAGAG<br>Coli-18:<br>GGGAAAUGUGGGCUCCCACAUACUCUGAUGAUCCUUCGGGAUCAUUCAUGGCAA	-47.68
+21	Broc+21:<br>GGGAAAUUGCCAUGUGUAUGUGGGAGACGGUCGGGUCCAGAUAUUCGUAUCUGUCGAGUAGAGUGU<br>Coli-21:<br>GGGAAAGGGCUCCCACAUACUCUGAUGAUCCUUCGGGAUCAUUCAUGGCAA	-47.18
+24	Broc+24:<br>GGGAAAUUGCCAUGUGUAUGUGGGAGACGGUCGGGUCCAGAUAUUCGU	-45.88

	Coli-24:<br>AUCUGUCGAGUAGAGUGUGGG<br>GGGAAACUCCCACAUACUCUGAUGAUCCUUCGGGAUCAUUCAUGGCA	
+27	Broc+27:<br>GGGAAAUUGCCAUGUGUAUGUGGGAGACGGUCGGGUCCAGAUAUUCGUAUCUGUC<br>GAGUAGAGUGUGGGCUC<br>Coli-27:<br>GGGAAACCACAUACUCUGAUGAUCCUUCGGGAUCAUUCAUGGCA	-41.88
+30	Broc+30:<br>GGGAAAUUGCCAUGUGUAUGUGGGAGACGGUCGGGUCCAGAUAUUCGUAUCUGUC<br>GAGUAGAGUGUGGGCUCCCA<br>Coli-30:<br>GGGAAACAUACUCUGAUGAUCCUUCGGGAUCAUUCAUGGCA	-43.08
+33	Broc+33:<br>GGGAAAUUGCCAUGUGUAUGUGGGAGACGGUCGGGUCCAGAUAUUCGUAUCUGUC<br>GAGUAGAGUGUGGGCUCCCACAU<br>Coli-33: GGGAAAACUCUGAUGAUCCUUCGGGAUCAUUCAUGGCA	-43.78
+36	Broc+36:<br>GGGAAAUUGCCAUGUGUAUGUGGGAGACGGUCGGGUCCAGAUAUUCGUAUCUGUC<br>GAGUAGAGUGUGGGCUCCCACAUACU+<br>Coli-36:<br>GGGAAACUGAUGAUCCUUCGGGAUCAUUCAUGGCA	-47.88
+39	Broc+39:<br>GGGAAAUUGCCAUGUGUAUGUGGGAGACGGUCGGGUCCAGAUAUUCGUAUCUGUC<br>GAGUAGAGUGUGGGCUCCCACAUACUCUG<br>Coli-39:<br>GGGAAAAUGAUCCUUCGGGAUCAUUCAUGGCA	-47.98
+42	Broc+42:<br>GGGAAAUUGCCAUGUGUAUGUGGGAGACGGUCGGGUCCAGAUAUUCGUAUCUGUC<br>GAGUAGAGUGUGGGCUCCCACAUACUCUGAUG<br>Coli-42:<br>GGGAAAAUCCUUCGGGAUCAUUCAUGGCA	-42.28
+45	Broc+45:<br>GGGAAAUUGCCAUGUGUAUGUGGGAGACGGUCGGGUCCAGAUAUUCGUAUCUGUC<br>GAGUAGAGUGUGGGCUCCCACAUACUCUGAUGAUC<br>Coli-45:<br>GGGAAACUUCGGGAUCAUUCAUGGCA	-39.18
+48	Broc+48:<br>GGGAAAUUGCCAUGUGUAUGUGGGAGACGGUCGGGUCCAGAUAUUCGUAUCUGUC<br>GAGUAGAGUGUGGGCUCCCACAUACUCUGAUGAUCCUU<br>Coli-48:<br>GGGAAACGGGAUCAUUCAUGGCA	-43.78
+51	Broc+51:<br>GGGAAAUUGCCAUGUGUAUGUGGGAGACGGUCGGGUCCAGAUAUUCGUAUCUGUC<br>GAGUAGAGUGUGGGCUCCCACAUACUCUGAUGAUCCUUCGG<br>Coli-51:<br>GGGAAAGAUCAUUCAUGGCA	-42.68
+54	Broc+54:<br>GGGAAAUUGCCAUGUGUAUGUGGGAGACGGUCGGGUCCAGAUAUUCGUAUCUGUC<br>GAGUAGAGUGUGGGCUCCCACAUACUCUGAUGAUCCUUCGGGAU<br>Coli-54:<br>GGGAAACAUUCAUGGCA	-43.38
+57	Broc+57:<br>GGGAAAUUGCCAUGUGUAUGUGGGAGACGGUCGGGUCCAGAUAUUCGUAUCUGUC<br>GAGUAGAGUGUGGGCUCCCACAUACUCUGAUGAUCCUUCGGGAUCAU<br>Coli-57:	-47.28

	GGGAAAUCAUGGCA	
-3	Broc-3:<br>GGGAAAUUGCCAUGUGUAUGUGGGAGACGGUCGGGUCCAGAU<br>Coli+3:<br>GGGAAAAUUCGUAUCUGUCGAGUAGAGUGUGGGCUCCCACAUACUCUGAUGAUCC<br>UUCGGGAUCAUUCAUGGCAA	-42.48
-6	Broc-6:<br>GGGAAAUUGCCAUGUGUAUGUGGGAGACGGUCGGGUCCA<br>Coli+6:<br>GGGAAAGAUAUUCGUAUCUGUCGAGUAGAGUGUGGGCUCCCACAUACUCUGAUGA<br>UCCUUCGGGAUCAUUCAUGGCAA	-46.68
-9	Broc:-9<br>GGGAAAUUGCCAUGUGUAUGUGGGAGACGGUCGGGU<br>Coli-9:<br>GGGAAACCAGAUAUUCGUAUCUGUCGAGUAGAGUGUGGGCUCCCACAUACUCUGA<br>UGAUCCUUCGGGAUCAUUCAUGGCAA	-46.98
-12	Broc-12:<br>GGGAAAUUGCCAUGUGUAUGUGGGAGACGGUCG<br>Coli+12:<br>GGGAAAGGUCCAGAUAUUCGUAUCUGUCGAGUAGAGUGUGGGCUCCCACAUACUC<br>UGAUGAUCCUUCGGGAUCAUUCAUGGCAA	-46.68
-15	Broc-15:<br>GGGAAAUUGCCAUGUGUAUGUGGGAGACGG<br>Coli+15:<br>GGGAAAUCGGGUCCAGAUAUUCGUAUCUGUCGAGUAGAGUGUGGGCUCCCACAUA<br>CUCUGAUGAUCCUUCGGGAUCAUUCAUGGCAA	-47.08
-18	Broc-18:<br>GGGAAAUUGCCAUGUGUAUGUGGGAGA<br>Coli+18:<br>GGGAAACGGUCGGGUCCAGAUAUUCGUAUCUGUCGAGUAGAGUGUGGGCUCCCAC<br>AUACUCUGAUGAUCCUUCGGGAUCAUUCAUGGCAA	-46.68
-21	Broc-21:GGGAAAUUGCCAUGUGUAUGUGGG<br>Coli+21:GGGAAAAGACGGUCGGGUCCAGAUAUUCGUAUCUGUCGAGUAGAGUG<br>UGGGCUCCCACAUACUCUGAUGAUCCUUCGGGAUCAUUCAUGGCAA	-42.38
-24	Broc-24:<br>GGGAAAUUGCCAUGUGUAUGU<br>Coli+24:<br>GGGAAAGGGAGACGGUCGGGUCCAGAUAUUCGUAUCUGUCGAGUAGAGUGUGGGC<br>UCCCACAUACUCUGAUGAUCCUUCGGGAUCAUUCAUGGCAA	-45.48
-27	Broc-27:<br>GGGAAAUUGCCAUGUGUA<br>Coli+27:GGGAAAUGUGGGAGACGGUCGGGUCCAGAUAUUCGUAUCUGUCGAGU<br>AGAGUGUGGGCUCCCACAUACUCUGAUGAUCCUUCGGGAUCAUUCAUGGCAA	-49.18
-30	Broc-30:<br>GGGAAAUUGCCAUGU<br>Coli+30:<br>GGGAAAGUAUGUGGGAGACGGUCGGGUCCAGAUAUUCGUAUCUGUCGAGUAGAGU<br>GUGGGCUCCCACAUACUCUGAUGAUCCUUCGGGAUCAUUCAUGGCAA	-49.18

Supporting Figures:

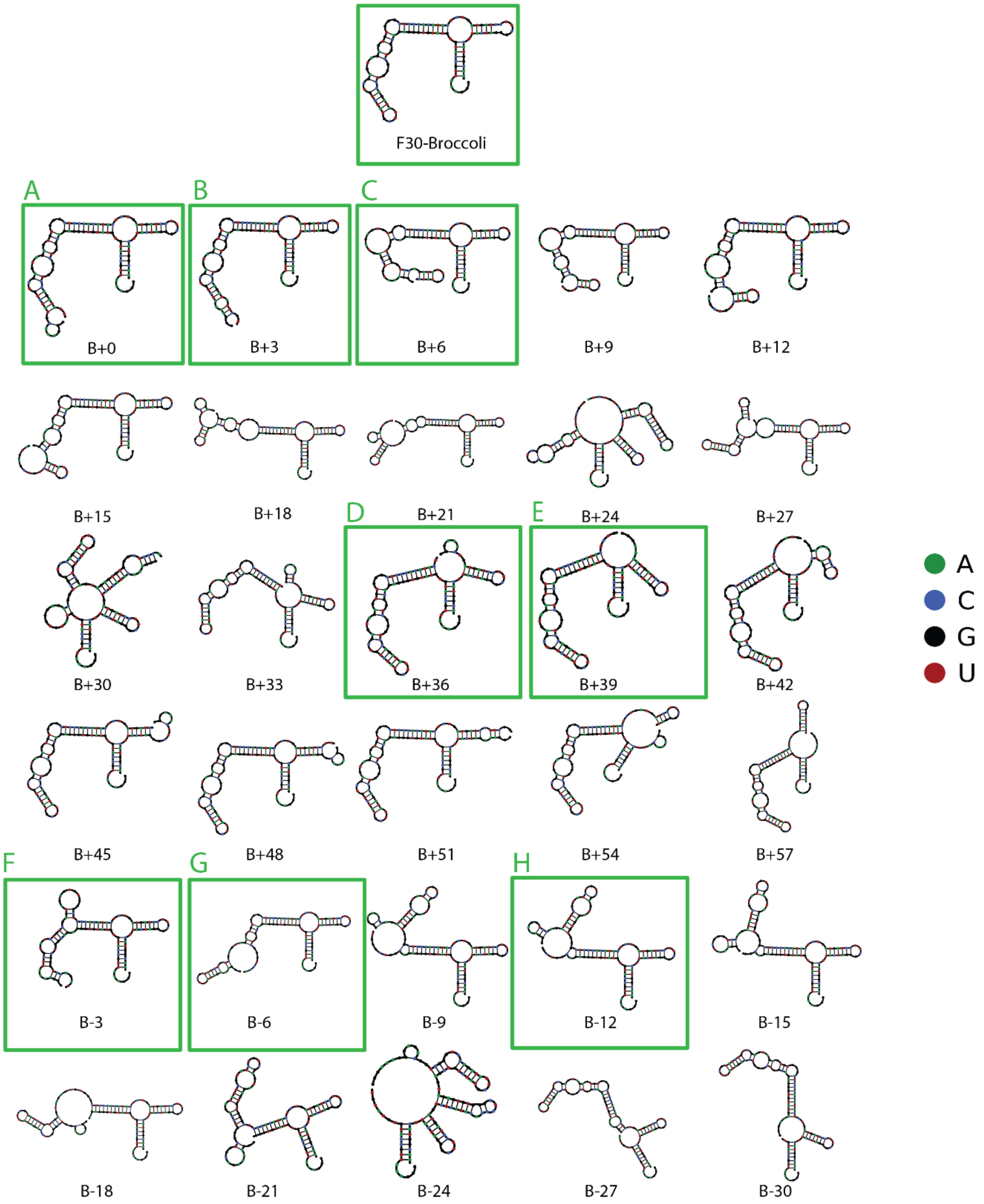

Supporting Figure S1: Secondary structures of designed Broccoli Fluorets, predicted by NUPACK[1] at 37 °C. Some of them are completely misfolded (e.g., B-24, B-27, etc) when compared to the secondary structure of original F30 Broccoli. Based on the secondary structure analysis, eight fluorets (boxed in green) were chosen for further experimental characterization. The splits of chosen constructs address the potential influence of different areas of F30-Broccoli aptamer on its fluorescent properties. The choice of ± 3 nts was arbitrary.

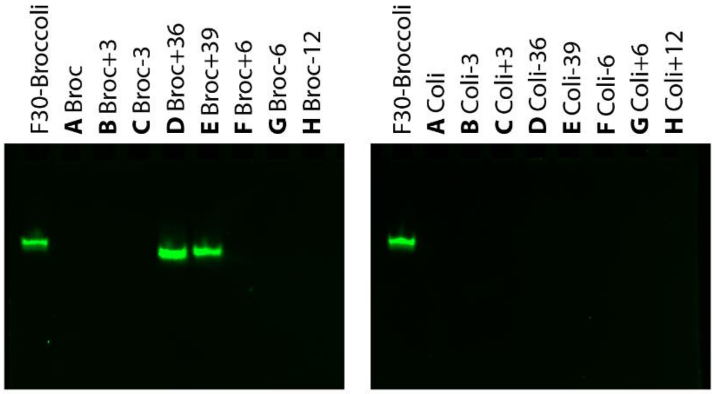

Supporting Figure S2: Fluorescence of individual monomer strands. Strands were visualized on an 8% native-PAGE stained with DFHBI-1T.

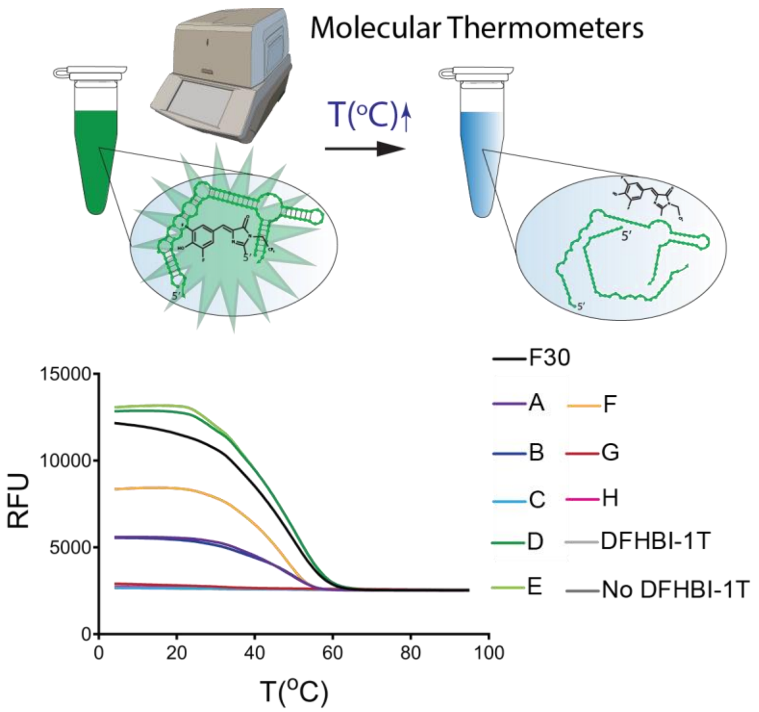

Supporting Figure S3: "Molecular Thermometers" that track the temperature changes via fluorescence deactivation.

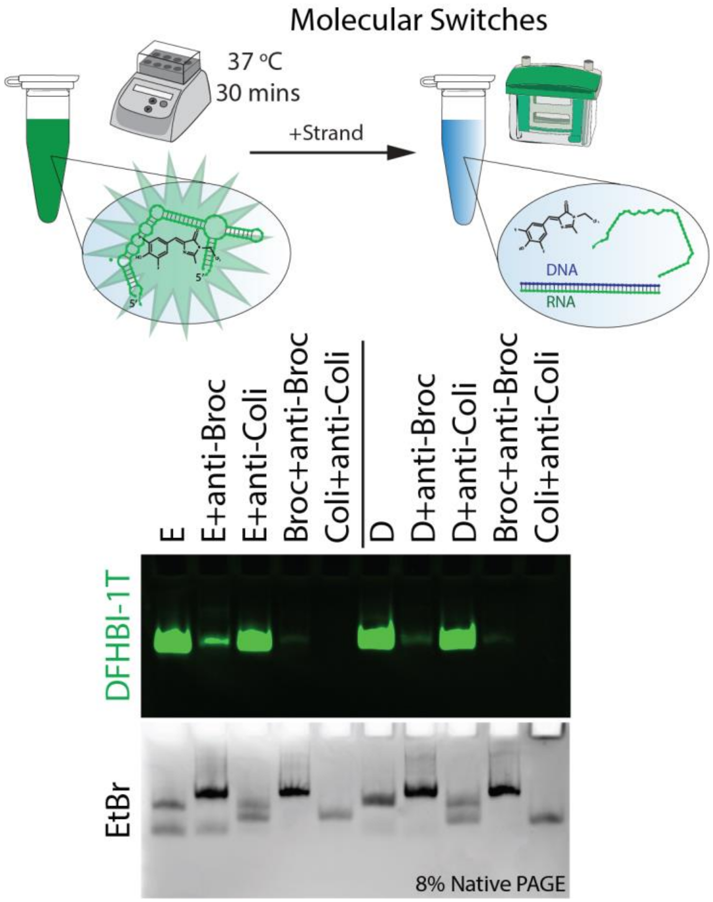

Supporting Figure S4: "Molecular Switches" responding to the introduction of oligonucleotides.

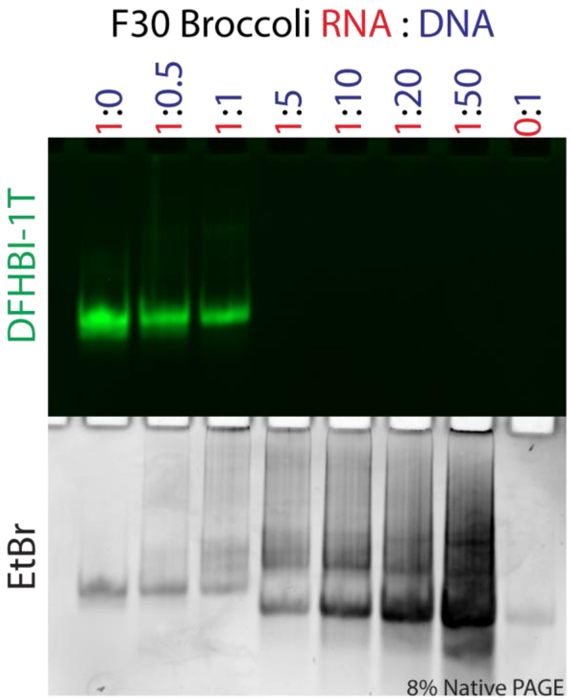

Supporting Figure S5: F30 Broccoli RNA assembled with increasing ratios of its complementary DNA. All RNAs are added at 1 μM.

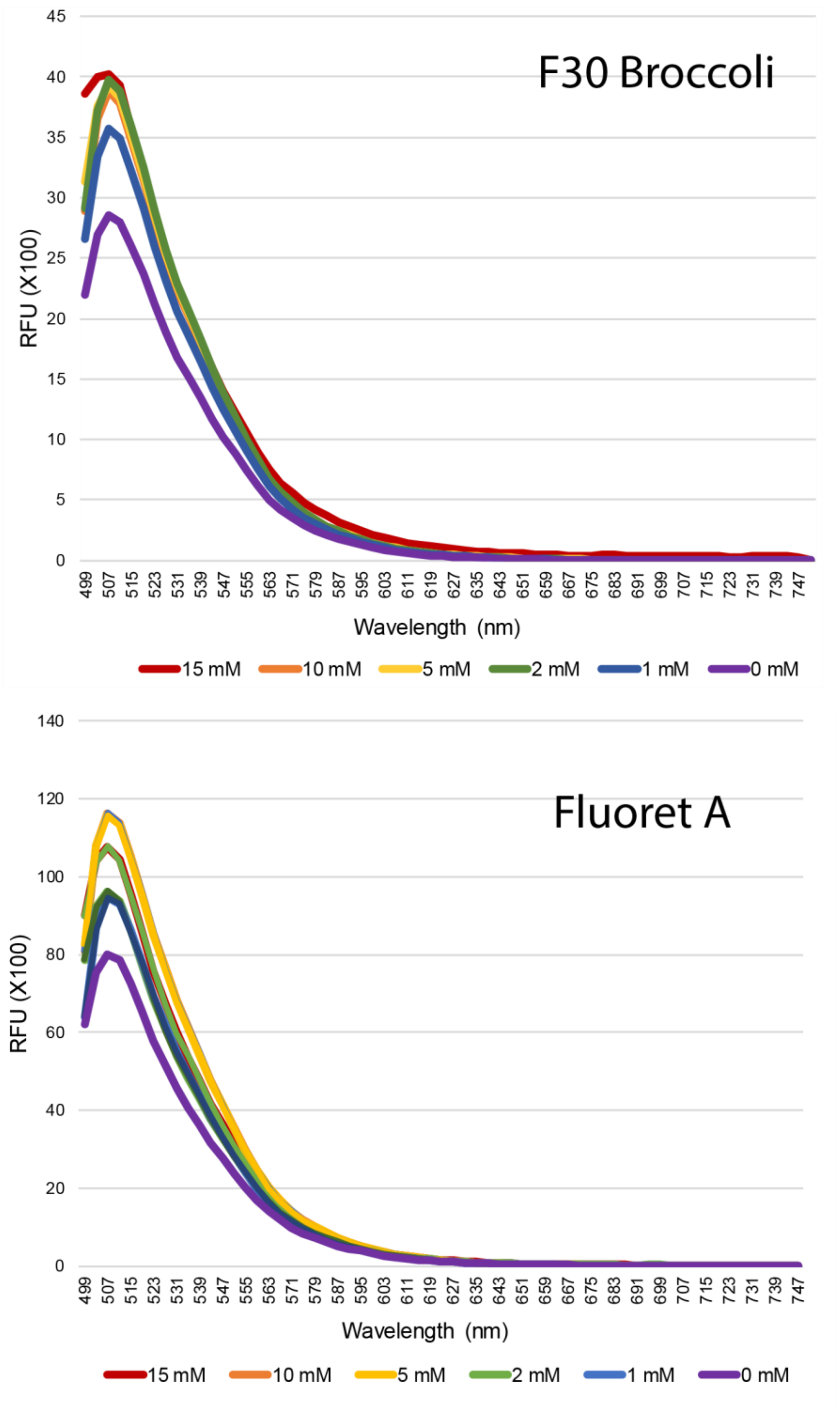

Supporting Figure S6: Assembly of F30-Broccoli aptamer and Fluoret A demonstrated at varying concentrations of Mg^{2+}.

	Strands in Fluoret	T_m (°C ± SEM)	$T_{1/2}$ (min)	RFU
	F30-Broccoli	44.02±0.34	2.49	29685.5
A	(Broc)+(Coli)	43.80±0.30	2.74	12857.3
B	(Broc+3)+(Coli-3)	43.51±0.29	2.81	4052.8
C	(Broc+6)+(Coli-6)	—	—	177.3
D	(Broc+36)+(Coli-36)	44.95±0.31	2.16	16507.0
E	(Broc+39)+(Coli-39)	44.57±0.13	2.44	26697.0
F	(Broc-3)+(Coli+3)	43.16±0.18	2.46	10401.2
G	(Broc-6)+(Coli+6)	—	—	95.7
H	(Broc-12)+(Coli+12)	—	—	42.2

Supporting Table S1. Physicochemical characterization of experimentally tested F30-Broccoli fluorets. Melting temperature (T_m) is presented ± SEM with n=3; Chemical stability ($T_{1/2}$) is presented with n=3; RFU is based on fluorescence spectra in Figure 1B.

References

1. Zadeh, J. N.; Steenberg, C. D.; Bois, J. S.; Wolfe, B. R.; Pierce, M. B.; Khan, A. R.; Dirks, R. M.; Pierce, N. A., NUPACK: Analysis and design of nucleic acid systems. *J Comput Chem* **2011,** *32* (1), 170-3.
